# Interaction of an atypical *Plasmodium falciparum *ETRAMP with human apolipoproteins

**DOI:** 10.1186/1475-2875-7-211

**Published:** 2008-10-20

**Authors:** Marissa Vignali, Anastasia McKinlay, Douglas J LaCount, Rakesh Chettier, Russell Bell, Sudhir Sahasrabudhe, Robert E Hughes, Stanley Fields

**Affiliations:** 1 Departments of Genome Sciences and Medicine, University of Washington, Box 355065, Seattle, WA 98195, USA; 2 Howard Hughes Medical Institute, USA; 3 Prolexys Pharmaceuticals, Inc., 2150 West Dauntless Avenue, Salt Lake City, Utah 84116, USA

## Abstract

**Background:**

In order to establish a successful infection in the human host, the malaria parasite *Plasmodium falciparum *must establish interactions with a variety of human proteins on the surface of different cell types, as well as with proteins inside the host cells. To better understand this aspect of malaria pathogenesis, a study was conducted with the goal of identifying interactions between proteins of the parasite and those of its human host.

**Methods:**

A modified yeast two-hybrid methodology that preferentially selects protein fragments that can be expressed in yeast was used to conduct high-throughput screens with *P. falciparum *protein fragments against human liver and cerebellum libraries. The resulting dataset was analyzed to exclude interactions that are not likely to occur in the human host during infection.

**Results:**

An initial set of 2,200 interactions was curated to remove proteins that are unlikely to play a role in pathogenesis based on their annotation or localization, and proteins that behave promiscuously in the two-hybrid assay, resulting in a final dataset of 456 interactions. A cluster that implicates binding between *P. falciparum *PFE1590w/ETRAMP5, a putative parasitophorous vacuole membrane protein, and human apolipoproteins ApoA, ApoB and ApoE was selected for further analysis. Different isoforms of ApoE, which are associated with different outcomes of malaria infection, were shown to display differential interactions with PFE1590w.

**Conclusion:**

A dataset of interactions between proteins of *P. falciparum *and those of its human host was generated. The preferential interaction of the *P. falciparum *PFE1590w protein with the human ApoE ε3 and ApoE ε4 isoforms, but not the ApoE ε2 isoform, supports the hypothesis that ApoE genotype affects risk of malaria infection. The dataset contains other interactions of potential relevance to disease that may identify possible vaccine candidates and drug targets.

## Background

Malaria is a devastating global disease, responsible for approximately 500 million clinical cases and more than a million deaths per year [1]. The most deadly form is caused by *Plasmodium falciparum*, which has a complex life cycle. The parasite is delivered to the human bloodstream as sporozoites that develop in the salivary gland of infected mosquitoes. These motile forms enter the liver, transversing through several cells before establishing themselves in a hepatocyte. Within the hepatocyte, the parasite undergoes a large number of asexual divisions, eventually releasing tens of thousands of merozoites that are capable of attaching to and invading erythrocytes. Within the erythrocyte, the parasite again divides several times in a 48-hour timeframe, releasing 16 or more merozoites that invade new erythrocytes. This intraerythrocytic stage is responsible for the cyclic symptoms of human disease (reviewed in [2]).

As a consequence of its life cycle, *P. falciparum *must be able to recognize, bind to and invade at least the two human cell types of hepatocytes and erythrocytes. These events are defined by particular molecular interactions. For example, hepatocyte invasion requires binding of the parasite's circumsporozoite surface protein to heparan sulfate proteoglycan surface receptors [3], although other *P. falciparum *proteins have been implicated in this process [4]. Many *P. falciparum *proteins have been proposed to be involved in erythrocyte invasion, and the human receptors for a few of them have been characterized (for example, EBA-175/glycophorin A and EBA-140/glycophorin C, MSP1/band3); however, the receptors for most of the parasite proteins implicated in erythrocyte invasion remain to be identified (reviewed in [5,6]).

In both hepatocytes and erythrocytes, the parasite is contained in a membranous structure known as the parasitophorous vacuole. Within this structure, however, the parasite is not isolated from the host cell, and must import nutrients from the plasma and the host cell cytoplasm to survive [7]. Particularly in the case of erythrocyte invasion, *P. falciparum *also establishes an active export of its proteins to the cytoplasm and surface of the host cell, where they modify the surface's structural and adhesive properties (reviewed in [8]). The presence of parasite proteins, such as PfEMP1 and other adhesins on the surface of erythrocytes, causes these infected cells to sequester to endothelia and placenta via interactions with surface receptor molecules such as CD36, chondroitin sulfate A, ICAM-1 and selectins, resulting in the evasion of these cells from the host immune system. In addition, several parasite proteins interact with human cytoskeletal proteins; for example, PfEMP3 interacts with spectrin and actin [9], and both RESA and KAHRP interact with spectrin [10,11].

Previously, a large network of interactions among the proteins of *P. falciparum *was generated [12]. The results of a similar high-throughput yeast two-hybrid analysis aimed at detecting interactions between proteins of *P. falciparum *and those of its human host are presented here. An initial dataset of over 2,200 putative interactions was curated to exclude interactions that are not likely to be relevant to the pathogenesis of the parasite, resulting in a final dataset of 456 interactions. Among these, a cluster of interactions involving *P. falciparum *PFE1590w/ETRAMP5 and human apolipoproteins ApoA1, ApoB and ApoE was studied further.

## Materials and methods

### Library construction and high-throughput yeast two hybrid screens

Protocols described in [12] were used to generate libraries of plasmids encoding *P. falciparum *and human protein fragments as fusions with Gal4 domains and auxotrophic markers to select for expression in yeast. High-throughput yeast two-hybrid screens, selection of positive colonies, PCR amplification and sequencing of inserts to determine the identity of the interacting partners were also performed following the techniques described in [12].

### Dataset curation and filtering

*Plasmodium falciparum *protein annotations were downloaded from PlasmoDB [13] and human protein annotations were downloaded from DAVID [14]. Information was also obtained from other databases, including GeneCards [15], Entrez Gene [16] and GeneDB [17]. K-clustering was performed as described in [12] and resulted in the exclusion of human proteins with 25 or more partners (k = 2) and *P. falciparum *proteins with 18 or more partners (k = 3).

### Two-hybrid retests

Plasmids were purified from the positive diploid yeast strains identified in the high-throughput screens, sequenced, and re-transformed into yeast. R2HMet yeast strains expressing different *P. falciparum *protein fragments as fusions with the *Saccharomyces cerevisiae *Gal4 DNA-binding domain and Met2 were mated against BK100 yeast strains expressing human protein fragments as fusions with the *S. cerevisiae *Gal4 activation domain and Ura3 (or its orthologue *S. pombe *Ura4) [12]. For retesting positives, diploids were selected in synthetic media lacking tryptophan and leucine with or without extra adenine, and their ability to activate the *HIS3 *reporter gene in the presence of 0 to 3 mM 3-aminotriazole was tested by streaking or serial dilution. Plates were grown at 30°C for up to a week and scanned.

### Protein extracts and western blots

Yeast extracts were prepared as described [18] and subjected to electrophoresis on 4–12% NuPAGE/MOPS gels (Invitrogen). Western blotting was performed using standard procedures with 1:100 dilution of anti-GAL4-TA (C-10) antibodies (Santa Cruz) and 1:5,000 dilution of anti-actin antibodies.

## Results

### Filtering of a high throughput two-hybrid dataset

To identify interactions between *P. falciparum *and human proteins, a modified yeast two-hybrid approach was applied as described previously [12]. This method preferentially selects for plasmids that encode fragments of proteins that can be expressed in yeast, and is particularly useful for *P. falciparum *proteins, which are difficult to express in heterologous systems. DNA-binding domain (BD) libraries of protein inserts from *P. falciparum *mRNA expressed during the intraerythrocytic cycle and activation domain (AD) libraries from human liver and cerebellum mRNA were generated. 9,408 two-hybrid screens with randomly chosen fragments of *P. falciparum *proteins were performed against the human activation domain libraries. Diploid yeast strains were selected and the identities of the putative interactive partners were determined as described [12]. These searches yielded 2,212 unique interactions involving 507 *P. falciparum *and 986 human proteins.

As with other high throughput two-hybrid studies, many of the putative interactions identified likely do not represent *bona fide *binding between a parasite and human protein but correspond instead to false positives that arise in these assays [19]. The bulk of these false positives are due to proteins that lead to transcriptional activation in yeast in the absence of any interaction between the proteins encoded by the two-hybrid vectors. Such proteins can be identified by a variety of means, including their over-representation among the putative interactions.

In other cases, the two-hybrid results reflect proteins that interact in the yeast assay but not in their normal *in vivo *setting. For example, the initial dataset contained examples of putative interactions between two orthologous proteins, one from *P. falciparum *and one from human, indicating most likely that each of these proteins normally homodimerizes. In other examples, a human protein was observed to interact with the *P. falciparum *orthologue of its known human protein partner. Both of these types of interactions reflect the high degree of conservation of these protein pairs between the two species.

Interactions more likely to be relevant to *P. falciparum *pathogenesis were identified by curation of the initial dataset, taking into account available information about the proteins involved in the interactions. Annotated and predicted GO classifications were considered, as well as other functions and properties of the proteins: the presence of transmembrane domains, evidence of membrane localization (except for nuclear membrane localization), and classification as extracellular or secreted protein were considered positive features. For *P. falciparum *proteins, the presence of a signal peptide, evidence or prediction of export to the host cell cytoplasm, presence in Maurer's clefts, localization to the surface of the infected erythrocyte, and classification as a putative adhesin were also considered as positive features (see PlasmoDB for data sources). Interactions involving *P. falciparum *or human proteins with likely nuclear function, mitochondrial localization, roles in basic cellular processes (including translation, RNA processing, protein degradation, ubiquitin metabolism and nuclear/cytoplasmic transport), and metabolic enzymes were excluded from the final dataset. Also excluded were interactions involving *P. falciparum *proteins annotated as chaperones, apicoplast or cytoskeleton proteins, if there was no additional evidence of their being exported to the host cell.

In addition, a large number of apparent partners that likely activate the two-hybrid reporter gene by mechanisms that do not involve *bona fide *interactions were eliminated by a k-clustering algorithm that takes into consideration the number of partners of each protein in the dataset to establish thresholds for exclusion. By this means, all proteins present as DNA-binding domain fusions with 18 or more interactions and as activation domain fusions with 25 or more interactions were excluded. Putative interactions of proteins that had been classified as promiscuous in previous large-scale yeast two-hybrid studies of *P. falciparum *proteins [12] or human proteins (unpublished data from Prolexys Pharmaceuticals, Inc.) were also excluded.

Although these filters potentially exclude some biologically relevant interactions, the majority of the interactions removed are most likely false positives. The resulting dataset consists of 456 interactions, involving 203 *P. falciparum *proteins and 314 human proteins (Additional file 1), and is enriched for proteins that are likely to be at the interface of the parasite and the host.

### Interaction of *P. falciparum *PFE1590w with human apolipoproteins

A cluster of interactions between *P. falciparum *hypothetical protein PFE1590w and human apolipoproteins ApoA1, ApoB and ApoE was identified from screens of the human liver cDNA library (Additional file 1). PFE1590w (alternatively known as etrampBLOB.1, ETRAMP5 and antigen 22) is a protein of unknown function that belongs to the ETRAMP (Early TRAnscribed Membrane Proteins) or SEP (Small Exported Proteins) family of single-pass transmembrane proteins [20,21]. Several ETRAMPs have been shown to localize to the parasitophorous vacuole, the membranous structure in which the parasite resides when inside the erythrocyte and hepatocyte [21]. A transfected myc-tagged version of PFE1590w localizes to the periphery of late ring and early trophozoite-infected erythrocytes, suggesting that, like other ETRAMPs, the native PFE1590w could also be localized to the parasitophorous vacuole membrane, where it would be at the interface between the parasite and the host cell [22]. Apolipoproteins are expressed by the liver and intestine and secreted to the plasma, where they bind cholesterol and participate in the clearance of lipoprotein particles by hepatocytes [23].

**Figure 1:**
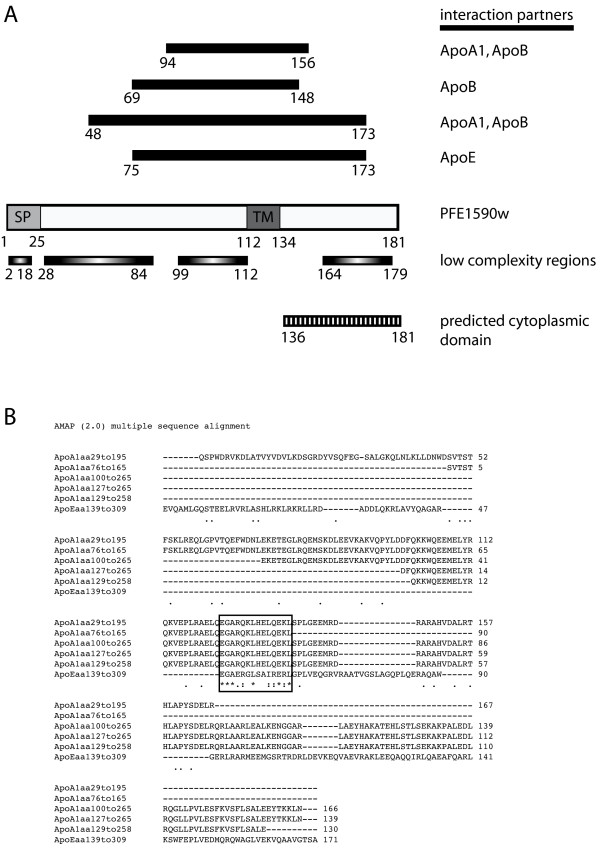
**Interactions between *P. falciparum *PFE1590w and human apolipoproteins ApoA, ApoE and ApoB identified by high-throughput screens**. (A) The fragments of PFE1590w involved in the interactions with human apolipoproteins are depicted above a diagram of the full-length protein, with the amino-terminal signal peptide (SP) and the transmembrane domain (TM) indicated. Low complexity regions are shown below. The human apolipoproteins that interacted with each PFE1590w peptide are listed to the right. The domain predicted by Phobius [30] to be exposed to the cytoplasm is shown. Numbers correspond to amino acid coordinates. (B) The fragments of ApoA1 and ApoE involved in the interactions were aligned by the MAVID/AMAP multiple alignment server [48]. The box indicates the region conserved among all ApoA1 and ApoE protein fragments identified in the screens.

Four overlapping fragments of PFE1590w interacted with 15 overlapping ApoA1 fragments, 1 ApoE fragment and 3 ApoB fragments (Figure 1A). All of the PFE1590w fragments contained the transmembrane domain and extended to different extents towards the amino- and carboxy-termini, including variable amounts of the region of highest amino acid complexity in the PFE1590w protein. ApoA and ApoE belong to the same family and share sequence and structural features, as well as common receptors on the surface of the hepatocytes [23]. Alignment of the fragments of ApoA and ApoE that interacted with PFE1590w revealed a conserved region that could be responsible for this interaction (Figure 1B).

**Figure 2:**
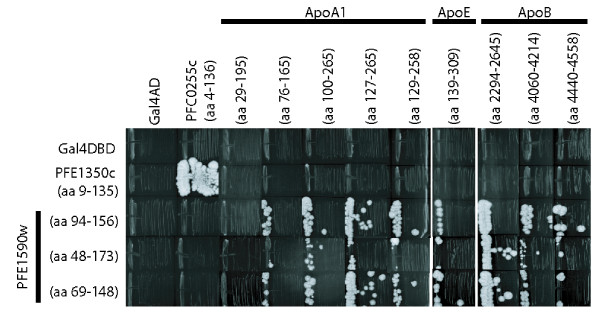
**Reproducibility and specificity of the *P. falciparum *PFE1590w interactions with human apolipoproteins ApoA, ApoE and ApoB**. Diploid strains expressing different combinations of PFE1590w and human apolipoprotein fragments as fusion proteins were tested for their ability to activate the *HIS3 *reporter gene in the presence of 3 mM 3-aminotriazole. The amino acid coordinates of the *P. falciparum *and human interacting protein fragments are indicated. Controls are strains carrying empty vectors encoding Gal4 BD or Gal4 AD, as well as fusions of Gal4 BD-PFE1350c (amino acids 9 to 135) and Gal4 AD-PFC0255c (amino acids 4 to 136), an interacting pair of *P. falciparum *proteins [12].

Plasmids encoding PFE1590w or apolipoprotein fragments were purified from yeast transformants and resequenced to determine both the exact starting and ending residues of the inserts as well as the reading frame with respect to the Gal4 DNA-binding or activation domain. Three of the PFE1590w fragments (excluding one that was highly similar to one of these three) were retested against nine different apolipoprotein fragments (all the ApoB and ApoE fragments, and five representative ApoA1 fragments) by introducing plasmids back into the yeast strains and repeating the mating and selection procedures in small scale. This assay confirmed binding between PFE1590w and ApoA1, ApoE and ApoB (Figure 2). The interactions were specific, as demonstrated by the lack of growth under selective conditions of strains carrying PFE1590w or an apolipoprotein insert along with an empty two-hybrid vector or one encoding for an unrelated fusion protein.

The gene for apolipoprotein ApoE is present in human populations as three alleles that differ only by the amino acid residue encoded in positions 112 and 158: ApoE ε2 contains cysteine in both positions, ApoE ε3 contains cysteine in position 112 but arginine in position 158, and ApoE ε4 has arginine in both positions [24]. These changes have been associated with differences in the structure of the protein, and in turn, their function in lipid metabolism [23]. Because the different ApoE alleles have been implicated in the outcome of malaria infection [25-27], the selectivity of interactions between the corresponding ApoE isoforms and *P. falciparum *PFE1590w was studied. To this end, full-length versions of the three alleles of ApoE (ApoE ε2, ε3, and ε4) were cloned into the Gal4 AD vector. To ensure that the interactions did not depend on the presence of *S. cerevisiae *Met2 on the carboxy-terminus of the PFE1590w fusion protein (which is used to select for expressed proteins in this version of the two-hybrid assay [12]), the *MET2 *coding sequence was replaced with that for glutathione S-transferase (GST). This construct yielded a stronger growth signal in the presence of the AD-apolipoprotein hybrids than the original one, and was, therefore, used for all further experiments.

**Figure 3:**
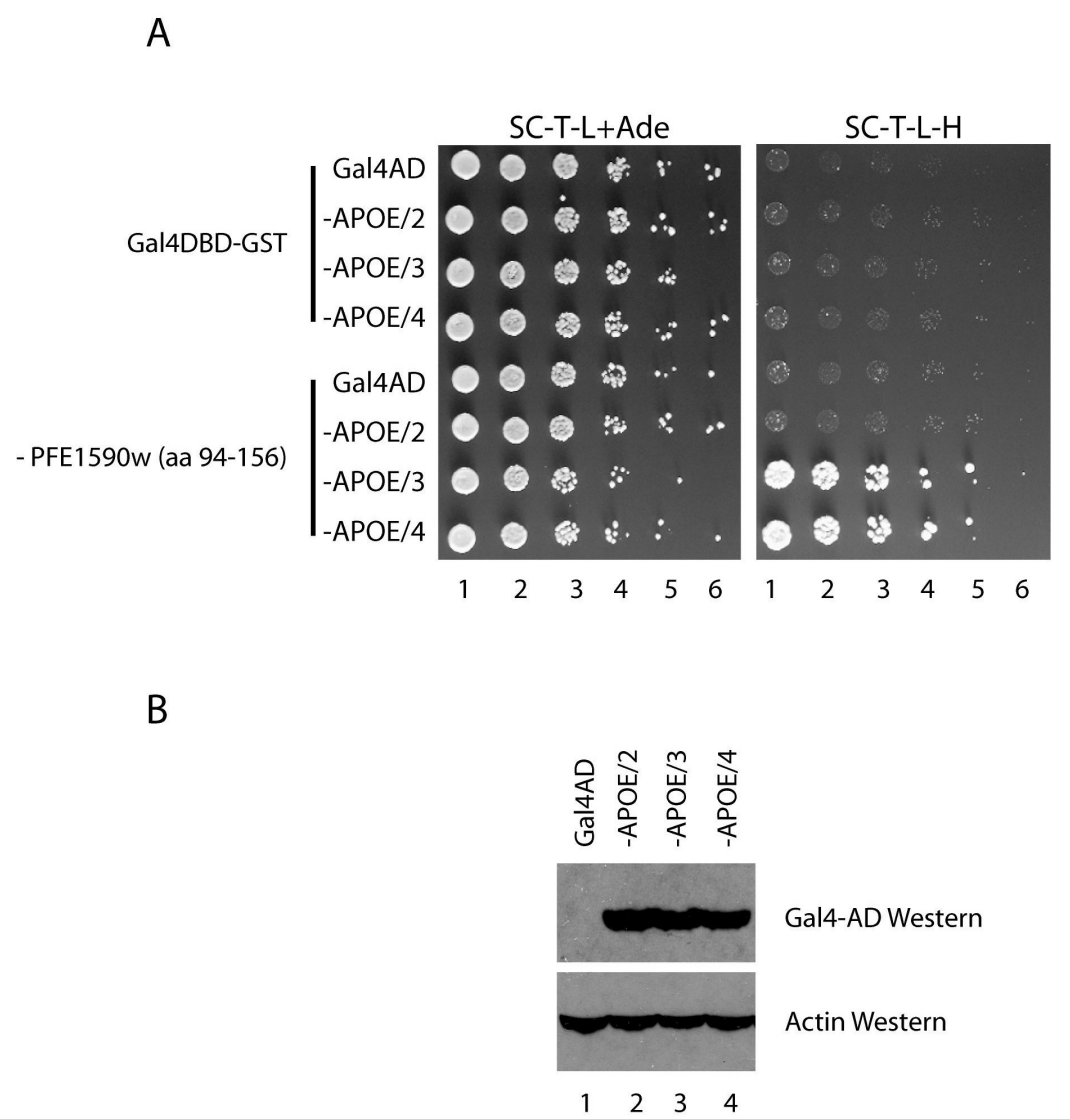
***P. falciparum *PFE1590w interacts differentially with ApoE isoforms ε2, ε3 and ε4**. (A) Yeast diploids containing the indicated plasmids were grown to an OD_660 _= 1.0, serially diluted and spotted onto solid synthetic complete media missing tryptophan and leucine and supplemented with extra adenine (SC-T-L+Ade), and onto plates missing tryptophan, leucine and histidine, and incubated at 30 C for 5–7 days. (B) Proteins were extracted from yeast cells grown in SC-T-L+Ade media to an OD_660 _= 1.0, separated by gel electrophoresis and transferred onto membranes by Western blot. Fusion proteins were detected with antibodies directed towards the Gal4 activation domain (upper panel), and antibodies against actin were used as a loading control (bottom panel).

Diploid yeast containing BD-PFE1590w (amino acids 94–156)-GST and an AD hybrid containing either of the two isoforms of the ApoE protein with arginine at position 158 (ApoE ε3 and ApoE ε4) grew well on selective media (Figure 3A, right panel). In contrast, when the yeast contained instead the ApoE ε2 isoform with cysteine at position 158, much weaker growth was observed. All transformants exhibited comparable growth on media that does not select for protein-protein interactions (Figure 3A, left panel). The ApoE selectivity is consistent with the results from the high-throughput screens that identified an ApoE fragment extending from residue 139 to 309 with arginine in position 158, corresponding to either the Apo ε3 or the ε4 isoform.

To rule out the possibility that differential two-hybrid signals could result from variable expression of the three ApoE isoforms in yeast, the levels of ApoE proteins were assayed by Western blot. Comparable expression of the three ApoE isoforms as AD hybrid proteins was observed (Figure 3B, top panel), with actin expression serving as a loading control (Figure 3B, bottom panel). Thus, these data indicate that the interaction of PFE1590w with ApoE proteins is dependent on the presence of arginine at position 158 of the ApoE protein. Polymorphisms at this position also influence the ability of ApoE to interact with other molecules, such as lipids and the LDL receptor [23].

## Discussion

Protein-protein interactions underlie the critical processes of infectious diseases, determining the specificity, affinity and efficiency by which pathogenic organisms are able to invade the human host. In the case of the malaria parasite *P. falciparum*, several processes that are essential to pathogenesis depend on host-parasite interactions, such as entry into host cells, growth and division within these cells, and binding to the lining of vasculature. An effort to identify such interactions was undertaken through the use of a large-scale yeast two-hybrid screening approach. The initial dataset of putative interactions was pared down to 456 interactions deemed most likely to be *bona fide *ones. However, two-hybrid false positives remain in this pared down set and no complementary verification of these interactions by a biochemical approach, such as co-immunoprecipitation, has been carried out; thus, these interaction data should be interpreted cautiously.

The interactions between *P. falciparum *hypothetical protein PFE1590w and human apolipoproteins ApoA1, ApoB and ApoE were further studied and determined to be specific by yeast two-hybrid. Furthermore, the preferential interaction of PFE1590w with the ApoE ε3 and ApoE ε4 isoforms but not the ApoE ε2 isoform was observed.

The genome of *P. falciparum *encodes 13 members of the ETRAMP protein family, which are predicted to have an amino-terminal signal peptide and a single transmembrane domain [21]. ETRAMPs are conserved among *Plasmodium *species, although PFE1590w does not have a clear orthologue in the rodent *Plasmodium *species. Unlike other ETRAMPs, PFE1590w is abundant in negatively charged residues, and has a 50-residue insertion rich in serines and prolines between the signal peptide and the transmembrane domain, resulting in its annotation as an atypical ETRAMP [21]. The gene encoding PFE1590w is expressed during the intraerythrocytic cycle [21,28,29] as well as in liver stages (Cate Speake and Patrick Duffy, personal communication). A transfected myc-tagged version of PFE1590w localizes to the periphery of the parasite in late ring and early trophozoite-infected erythrocytes, suggesting a parasitophorous vacuole membrane localization for this protein inside the infected erythrocytes [22]. Phobius [30], a programme that predicts the orientation of membrane proteins, predicts that residues 136–181 are cytoplasmic (Figure 1A), suggesting that the carboxy-terminal domain involved in the interactions described in this study could be exposed to the cytoplasm of the host cell. Since apolipoprotein-containing particles are internalized by the hepatocyte [23], if PFE1590w also localizes to the parasitophorous vacuole during the liver stages, the interactions between PFE1590w and apolipoproteins could take place in the hepatocyte.

The mRNA for PFE1590w has also been detected in gametocytes and in sporozoites [21,29], and PFE1590w peptides have been detected by mass spectrometry in trophozoites and in the membrane of the infected erythrocytes [31,32]. Parasite inhibitory antibodies against PFE1590w are present in the sera of individuals that have suffered malaria infections, supporting the idea that PFE1590w might be a surface protein, although it is also possible that this immunity is a result of exposure of the immune system of the host to the protein upon lysis of the parasite [33]. Therefore, besides being localized to the parasitophorous vacuole membrane, PFE1590w could be present on the plasma membrane of the sporozoite or the membrane of the infected red blood cell, where it could interact with plasma apolipoproteins.

Other ETRAMPs have been shown to be involved in interactions with host proteins. *Plasmodium yoelii *UIS3, a rodent ETRAMP essential for parasite development during liver stages that is orthologous to *P. falciparum *PF13_0012, interacts with liver fatty-acid binding protein (L-FABP) [34]. UIS3 localizes to the parasitophorous vacuole membrane within the hepatocyte [35], and binds L-FABP via its carboxy-terminal domain, which is predicted to be exposed to the cytoplasm of the host cell cytoplasm. Down-regulation of L-FABP by RNAi greatly inhibits the growth of parasites in cultured hepatoma cells, whereas overexpression of L-FABP promotes growth [34]. An interaction between human L-FABP and *P. falciparum *PFD0090c was observed in this study. PFD0090c is a hypothetical protein of the pHIST family[36], and is predicted to be exported to the cytoplasm of the host cell. In addition, PFD0090c has also been found by mass spectrometry on the surface of the infected erythrocyte [31].

Several lines of evidence suggest that apolipoproteins might have a role in the pathogenesis of the malaria parasite. First, apolipoproteins have been reported to inhibit invasion of hepatocytes by *Plasmodium *sporozoites by competing with the most abundant protein on the surface of sporozoites, circumsporozoite protein, for binding to HepG2 cultured liver cells, and delaying liver-mediated clearance of circumsporozoite protein from circulation [37]. Second, apolipoprotein E-enriched β-very low density lipoprotein inhibits invasion of HepG2 cells by sporozoites of a rodent *Plasmodium *species, *P. berghei*, and mice with high levels of circulating apolipoproteins have lower hepatic parasite loads. Based on this evidence, Sinnis *et al. *[37] postulated that invasion of hepatocytes by the parasite and lipoprotein uptake by the liver share a common mechanism that is likely mediated by binding to heparan sulfate proteoglycans, the physiological receptors of apolipoproteins on the surface of the hepatocytes. Third, studies of human populations have revealed correlations between the apolipoprotein E genotype of the human host and the susceptibility to malaria infection; although the ApoE ε3 allele is the most frequent worldwide, the ApoE ε4 allele, which is possibly the ancestral one, has an extremely high frequency in malaria endemic areas, including sub-Saharan Africa and Papua New Guinea [38,39]. In addition, Gambian children homozygous for the ApoE ε2 allele are more likely to suffer early malaria infection [25], while heterozygotes carrying the ApoE ε3 and ε4 alleles are more likely to suffer severe malaria, including cerebral malaria and severe anaemia [26]. These apparently contradictory data are compatible with the notion that children who suffer infections earlier in life develop protection against severe malaria [27]. While these results are based on a small number of individuals, they suggest that the ApoE genotype of the human host influences the outcome of malaria infection. The data presented here that indicates a selective interaction between PFE1590w and ApoE alleles ε3 and ε4 is consistent with the notion that this interaction might be related to malaria pathogenesis. It is possible that the PFE1590w – ApoE interaction is important for cerebral malaria and severe anaemia, and thus individuals carrying ApoE ε3 and ε4 alleles are more likely to develop these symptoms.

The relevance of the PFE1590w – apolipoprotein interaction for the parasite's ability to infect or develop within human host cells remains to be determined. Apolipoproteins are synthesized in the liver, and are cleared from circulation by the liver. The interaction may be involved in the invasion of liver cells by sporozoites, or alternatively, the interaction could be important for the parasite's survival inside the red blood cell or the hepatocyte. As in the case of the *P. yoelii *UIS3 – L-FABP interaction, PFE1590w might be involved in the uptake of lipids, in particular cholesterol, from the host. *Toxoplasma gondii*, an apicomplexan parasite related to *P. falciparum*, critically depends on host cholesterol from low-density plasma lipoproteins for survival, which it acquires by co-opting the host endocytosis pathway and sequestering lysosomes into its parasitophorous vacuole [40-42]. Although the proteins responsible for cholesterol acquisition in *T. gondii *have not been identified, it is likely that they are also localized to the parasitophorous membrane [40-42]. Thus, both *P. falciparum *and *Toxoplasma gondii *might have similar mechanisms of nutrient acquisition from the host cell.

Other interactions between *P. falciparum *and human proteins that may shed light on parasite invasion and other processes were identified in this study. For example, in a screen against the liver human library, an interaction was observed between a fragment of *P. falciparum *EXP-1 (PF11_0224) and overlapping fragments of human Programmed Cell Death 6-Interacting protein (PDCD6IP, or ALIX) (Additional file 1). This interaction was shown to be reproducible and specific by yeast two-hybrid (data not shown). EXP-1 is a well-characterized integral membrane protein localized to the parasitophorous vacuole [43,44]. PDCD6IP/ALIX is a class E vacuolar sorting protein involved in the transport of cargo proteins by the multivesicular body for incorporation into intralumenal vesicles; fusion between endosomes and the vacuole results in the localization of cargo proteins to the vacuolar lumen [45]. In addition, PDCD6IP/ALIX is used by several viruses, including HIV-1, for exiting infected cells [46]. Although the interaction was identified in the high-throughput screens against the liver library, EXP-1 is also expressed in blood stages, and PDCD6IP/ALIX has been found by mass spectrometry in the cytoplasm of the erythrocyte [47], suggesting that the interaction could take place in either the hepatocyte or the erythrocyte. It is possible that this interaction is involved in acquisition of vesicle contents by the parasite.

## Conclusion

More than 450 putative interactions between *P. falciparum *proteins expressed during the intra-erythrocytic cycle and human proteins expressed in liver or cerebellum are presented. The interaction between *P. falciparum *PFE1590w, an ETRAMP, and human apolipoproteins was further studied, and a differential interaction between PFE1590w and isoforms of ApoE that have been associated with different outcomes of malaria infection was observed. It is likely that this dataset will include other interactions of potential interest that will expand the understanding of the interplay between the parasite and the human host and may lead to the identification of potential vaccine candidates and drug targets.

## Competing interests

The authors declare that they have no competing interests.

## Authors' contributions

MV participated in the generation of the *P. falciparum *Y2H libraries and data analysis and curation, and drafted the manuscript. AM retested the interactions and carried out the Western blots. DJL participated in the generation of the *P. falciparum *Y2H libraries and data analysis. RC and RB managed the database of interactions. REH, SS and SF conceived the study and participated in its coordination. All authors read and approved the final manuscript.

## Supplementary Material

Additional file 1 Curated list of interactions identified between *Plasmodium falciparum *and human protein fragments. Click here for file
